# Hypovitaminosis D among newly diagnosed pulmonary TB patients and their household contacts in Uganda

**DOI:** 10.1038/s41598-022-09375-7

**Published:** 2022-03-28

**Authors:** Ester Lilian Acen, Irene Andia Biraro, Mudarshiru Bbuye, David Patrick Kateete, Moses L. Joloba, William Worodria

**Affiliations:** 1 Department of Physiology, School of Biomedical Sciences, College of Health Sciences, Makerere University, Kampala, Uganda; 2 Department of Internal Medicine, School of Medicine, College of Health Sciences Unit Makerere University, Kampala, Uganda; 3 Medical Research Council/Uganda Virus Research Institute and London School of Hygiene and Tropical Medicine Uganda Research Unit, Entebbe, Uganda; 4 Makerere Lung Institute College of Health Sciences Makerere University, Kampala, Uganda; 5 Department of Immunology and Molecular Biology, School of Biomedical Sciences, College of Health Sciences, Makerere University, Kampala, Uganda; 6 Pulmonary Division, Department of Medicine, Mulago National Referral Hospital, Kampala, Uganda

**Keywords:** Immunology, Microbiology, Physiology, Diseases

## Abstract

An estimated one billion people globally live with hypovitaminosis D. Studies have indicated that vitamin D deficiency is a risk factor for active tuberculosis (TB) disease. The aim of this study was to determine the association between vitamin D deficiency and TB status among patients with active TB, latent TB infection (LTBI) and those without TB infection. In a cross-sectional study of active TB patients, LTBI, QuantiFERON GOLD testpositive and (QFN^+^TST^+^) household contact and controls QuantiFERON GOLD testnegative (QFN^−^TST^−^) samples vitamin D levels were compared. Vitamin D status was determined by measurement of total vitamin D levels with 56 samples of active TB patients, 17 with LTBI, and 22 without TB infection using electrochemiluminescence. The median interquartile range (IQR) age of the study participants was 28 (20–35) years, and the majority (63%) were females. The median (IQR) vitamin D levels were 18 ng/ml (14–24). All groups had vitamin D hypovitaminosis with significantly lower levels among active TB patients (17 ng/ml, 13, 2) than among LTBI individuals (23 ng/ml 16–29) and those without TB infection (22 ng/ml, 17–28).

## Introduction

Vitamin D deficiency is predominant across all ages, with an estimate of approximately a billion people globally living with hypovitaminosis D^1,2^. Both industrialized and developing countries have reported deficient vitamin D in their population, yet it is a known risk factor for poor immune systems^3–7^. Over the years, evidence has shown that the vitamin D pathway plays a role in the immune system, where immune cells, including macrophages, augment antimicrobial peptide expression, which regulates the inflammatory response^8,9^. Cathelicidin antimicrobial peptide is a defense molecule that is involved in both innate and adaptive immunity and modulates infections through Toll-like receptor responses^10^. Through its expression, cathelicidin aids in restraining the growth of intracellular *Mycobacterium tuberculosis*^11,12^. Several studies have provided evidence that vitamin D deficiency is a risk factor for active TB disease^13–16^. A few other studies have reported low vitamin D levels in latent TB individuals compared to their controls^17,18^. Furthermore, studies have found that TB patients have lower vitamin D levels than other contacts from the same population^4,19,20^. Similarly, according to a meta-analysis, vitamin D supplementation affected sputum conversion and was therefore recommended for consideration during TB therapy^21^. In contrast, another meta-analysis confirmed no relationship between vitamin D supplementation and sputum conversion^22^.

Although the free and bioavailable vitamin D levels penetrate the cell and are involved in biological activities, they have not yet been used to define vitamin D status. Total 25-hydroxyvitamin D_,_ also known as 25(OH)D, is accepted as the best indicator for vitamin D status, as defined by the Institute of Medicine (IOM) and the Endocrine Society, and this has been adopted by several studies^11,12,23–29^. However, inconsistent findings on vitamin D status have been reported from various regions^30,31^. A study from Tanzania noted inconsistencies due to the selection bias of the control group^32^. On the other hand, according to our previous systematic review and meta-analysis, these inconsistencies are attributed to the variable definition of vitamin D deficiency^33^. Notably, the present study used the definition of the Endocrine Society for clear categorization of deficient and insufficient groups.

Furthermore, evidence of hypovitaminosis D was recognized among TB patients much earlier, as reported by Davies et al.^34^. In Uganda, a high prevalence of vitamin D deficiency (44.2%) was reported among hospitalized adult pulmonary TB patients^35^. The previous study did not include control groups for comparison of vitamin D status. This study aimed to determine vitamin D deficiency among active TB patients, LTBI individuals, and those without TB infection and to determine its association with TB status. The study findings may increase the scope of knowledge on the role of vitamin D and TB status, which may be important in the diagnosis or therapy of TB.

## Results

### Baseline characteristics of study participants

A total of 105 serum samples from patients were included in the analyses. Of these, 10 samples had undetectable values of vitamin D on analysis and were therefore removed. A total of 95 samples in the present study were from 56 active TB patients, 17 LTBI individuals, and 22 individuals without TB infection. The median (IQR) age of the participants was 28 (20–35) years. The majority of the participants (60/105, 63%) were females. Twenty-one percent of the study participants were HIV positive. Table 1 shows details of other social and demographic characteristics of the study population. At least 42% of participants reported having daily sunshine exposure between 1 and 7 h daily according to the sunshine exposure assessment. According to the vitamin D nutritional assessment, between 17 and 51% of the TB patients did not have a diet that contained vitamin D, which included milk, soya, liver, eggs, mushroom, and other nutrients.

**Table 1:** Baseline sociodemographic characteristics of participants.

Participant characteristic	Frequency (n/%)	Median (IQR)
**Age (years)**		28 (8, 65)
18 and below	16 (17)	
19–30	40 (42)	
31–40	22 (23)	
Above 40	17 (18)	
**Sex**		
Female	60 (63)	
Male	35 (37)	
**TB status**		
No TB infection	22 (23)	
Latent TB infection	17 (18)	
Active TB	56 (59)	
**Occupation**		
Business	26 (27)	
Skilled employment	11 (12)	
Unemployed	37 (39)	
Unskilled employment	21 (22)	
**Alcohol consumption**		
No	66 (71)	
Yes	27 (29)	
**Smoking**		
No	80 (86)	
Yes	13 (14)	
**HIV status**		
Negative	74 (79)	
Positive	20 (21)	

n is the number of variables with their percentages and IQR is the interquartile range of the median.

### Vitamin D levels among TB patients, LTBI individuals, and those without TB infection

**Figure 1:**
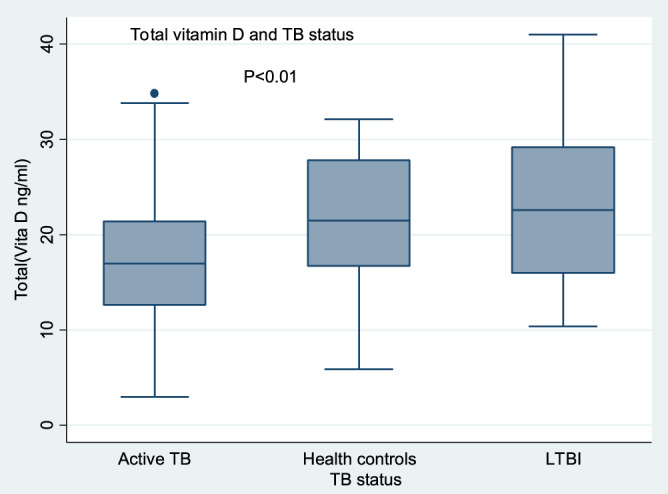
Box plot of vitamin D levels in participants with ATB, LTBI, and without TB infection. The boxes represent medians, and the upper and lower ends of the box represent the 75th and 25th percentiles, while the whiskers are the 5th and 95th percentiles with significance at < 0.05. Low vitamin D levels were observed in all three study groups with the lowest levels found in active TB patients (< 20 n/ml). The figure was generated using STATA version12, https://www.stata.com by M.B.

**Figure 2:**
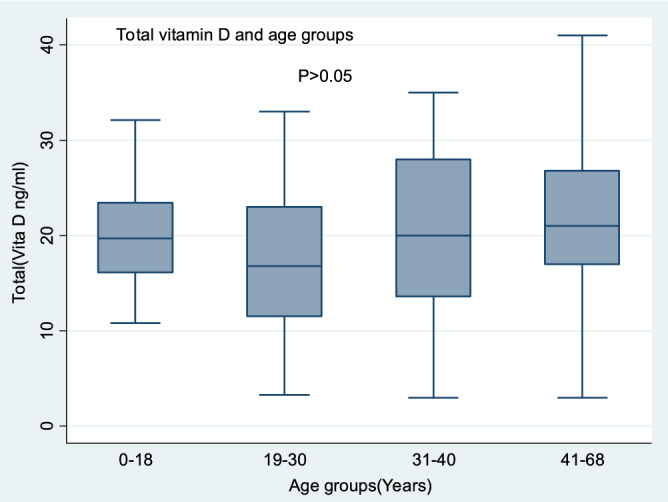
Vitamin D levels and age of active TB patients, LTBI individuals, and those without TB. The boxes represent medians, and the upper and lower ends of the box represent the 75th and 25th percentiles, respectively, while the whiskers are the 5th and 95th percentiles for significance at < 0.05. All ages show deficient and insufficient median levels with no optimal median levels (≥ 30 ng/ml) recorded. The figure was generated using STATA version12, https://www.stata.com by M.B.

The overall median (IQR) vitamin D levels of the participants were 18 ng/ml^14–24^. We found a significant difference in the median (IQR) vitamin D levels among the three groups; active TB patients 17 ng/ml^13,21^ compared to LTBI individuals 23 ng/ml^16,29^ and those without TB infection were 22 ng/ml^17,28^. Table 2 and Fig. 1 show the details. The maximum levels were 41 ng/ml, and the lowest was < 3.0 ng/ml. A prevalence of 52 (55%) vitamin D deficiency (< 20 ng/ml) was noted among the study participants, with 13% severe vitamin D deficiency (< 10 ng/ml) and only 9 (9.5%) with optimal levels (≥ 30 ng/ml). Hypovitaminosis D was noted across all ages with no significant difference (Table 2 and Fig. 2). There was no significant difference between vitamin D levels among smokers and nonsmokers (Table 2). Severe vitamin D deficiency was predominantly found among TB patients and not among LTBI individuals. However, 2 participants with severe vitamin D deficiency were found in the group with those without TB infection. Overall, we found a high prevalence of hypovitaminosis D (88%) among our study participants. Table 3 shows details of vitamin D status. We further analysed the participant characteristics according to vitamin D status, but no significant difference was observed in any of them, as shown in Table 4. Analysis by age did not reveal any significant difference, as shown in Fig. 2. Furthermore, a subanalysis was performed to determine the association between total vitamin D levels and age, and a very weak positive association was found (rho 0.2, p = 0.04). Analysis between HIV status and total vitamin D in the three groups revealed significantly lower vitamin D levels among the active TB patients compared to other groups among both HIV-negative and HIV-positive individuals. We did not have HIV-positive individuals among the group of those without TB infection.

**Table 2:** Median differences in total vitamin D among participant characteristics.

Participant characteristics	Median (IQR) total vitamin D	p -value
**Sex**		0.45
Female	20 (15, 25)	
Male	17 (13, 23)	
**Age**		0.42
18 and below	20 (16.15, 23.45)	
19–30	17 (11.5, 23)	
31–40	20 (13.6, 28)	
Above 40	21 (17, 26.8)	
**Alcohol**		0.20
No	18 (13.6, 22.9)	
Yes	21 (13.8, 28)	
**BCG scar**		0.89
No	18 (15, 23)	
Yes	18 (13, 24)	
**HIV status**		0.7
Negative	17 (15, 24)	
Positive	19 (10.2, 25.1)	
**TB status**		< 0.001
Active TB	17 (12.6, 21.4)	
No TB infection	22 (16.7, 27.8)	
Latent TB infection	23 (16, 29.2)	
**Total vitamin D levels**		< 0.001
Very deficient	6 (4, 7)	
Deficiency levels	16 (14, 17)	
Insufficiency	23 (22, 28)	
Sufficient/optimal	34 (32, 35)	

IQR is interquartile range, vitamin D levels are in ng.ml and p value < 0.05.

**Table 3:** Vitamin D status in study participants with TB, latent TB, and without TB infection.

Category	TB n (%)	LTBI n %	Without TB n (%)	Overall status (%)
Deficiency	26 (65%)	7 (17%)	7 (17%)	42
Insufficiency	17 (50%)	6 (118%)	11 (32%)	36
Optimal	3 (33%)	4 (44%)	2 (22%)	10
Severe vitamin D deficiency	10 (83%)	0 (0%)	2 (17%)	13
**Other categories**				
Hypovitaminosis D	51 (53%)	22 (24%)	8 (9%)	85

Severe vitamin D levels < 10 ng/ml vitamin D deficiency < 20 ng/ml insufficiency = 21–29 ng/ml and optimal levels =  ≥ 30 ng/ml.

**Table 4:** Participant characteristics according to vitamin D levels.

Variables	Severe deficiency n (%) Median (range)	Deficiency n (%) Median (range)	Insufficient n (%) Median (range)	Sufficient n (%) Median (range)	p-value
Age (years)	27 (19, 45)	25 (10, 60)	29 (8, 58)	33 (17, 65)	0.16
**Sex**					
Female	8 (67)	22 (55)	24 (71)	6 (67)	0.57
Male	4 (33)	18 (45)	10 (29)	3 (33)	
**Alcohol consumption**					0.57
No	8 (67)	30 (77)	23 (70)	5 (56)	
Yes	4 (33)	9 (23)	10 (30)	4 (44)	
**BCG scar**					0.99
No	5 (42)	18 (46)	14 (42)	4 (44)	
Yes	7 (58)	21 (54)	19 (58)	5 (56)	
**HIV status**					0.30
Negative	7 (58)	33 (85)	27 (79)	7 (78)	
Positive	5 (42)	6 (15)	7 (21)	2 (22)	

Severe deficiency < 10 ng/ml, deficient ≤ 20 ng/ml, insufficient 21–29 ng/ml, sufficient ≥ 30 ng/ml.

### Association between total 25(OH) D levels and TB status

A weak negative association was found between active TB and vitamin D levels (Table 3). A post hoc analysis was performed to determine the exact significant difference using the Mann–Whitney test, and a significant difference was found between active TB and those without TB infection and with active TB with LTBI. Table 5 presents the details of this analysis.

**Table 5:** Association of total 25(OH) D and TB status.

Variables	Coefficient	95% confidence interval	p -value
**Total 25(OH)D**			
Control	Reference		
LTBI	1.6	− 3.2 to 6.5	0.51
Active TB	− 4.9	− 8.7 to − 1.2	0.01

p value = 0.05 is significant.

## Discussion

Vitamin D is involved in innate and adaptive immunity, and vitamin D deficiency has been identified as a risk factor for TB disease^36,37^. The present study reports high hypovitaminosis D (88%) among the study population and vitamin D deficiency of 55%. Among the active TB group alone, the prevalence of vitamin D deficiency was 66%. This prevalence is higher than that in a previous Ugandan study that reported a prevalence of 44.2% vitamin D deficiency in admitted pulmonary TB patients; however, this study did not have controls^35^. Notably, our study reported lower vitamin D levels (17 ng/ml) than those in a previous study, which reported 23 ng/ml. Furthermore, a significant difference was found between the active TB patients compared to the LTBI and those without TB infection. Notably, however, all study groups had low vitamin D levels. This is an indication of a high prevalence of vitamin D insufficiency among the Ugandan study population. Our findings are closer to those of a study in South Africa among blacks with active TB disease and LTBI^38^. In this study, vitamin D deficiency was highly prevalent in all study groups. Comparable to our findings were active TB patients who had low vitamin D deficiency compared with their controls in studies from India, Ethiopia Egypt, Sudan, Iran, and Pakistan^20,29,39–42^. Contrary to our study, a previous study by Ho-Pham et al. from Vietnam reported no significant difference between vitamin D levels among TB patients and non-TB controls^43^. On the other hand, findings from the Tanzania study reported higher vitamin D levels in TB patients than in the non-TB controls^32^.

According to the author of that study, this finding of elevated 25(OH)D in PTB patients was due to alpha(1)-acid glycoprotein (AGP), a slow acute phase protein reactor that positively correlated with the metabolite^44,45^.

Smoking is a known risk factor for TB; however, we did not find a significant difference in vitamin D status between smokers and nonsmokers. Smoking affects the metabolism of vitamin D and alters calcium in the intestines^46^. An earlier study attributed this result to the elevation of CYP24A1 (24-hydroxylase) involved in the degradation of 1,25(OH)D3 or reduction in decreasing CYP27B1 (1α hydroxylase), which activates the production of 1,25(OH)D3^47^.

HIV infection enhances susceptibility to TB infection and has been recognized as a risk factor for vitamin D deficiency; therefore, we performed an analysis to determine the relationship between HIV and vitamin D status among the three groups. No significant difference was observed in vitamin D levels in our study. This finding may be compared to a study among the black population in South Africa that found vitamin D deficiency in both HIV-infected and non-infected individuals^38^. Our finding is similar to a report from a meta-analysis by Haung et al. in Africans that did not show a significant difference between HIV-positive and HIV-negative individuals^36^.

Regarding age, we did not find any age group with sufficient vitamin D levels, and no significant difference was found in vitamin D levels across the age groups. This finding is different from the Ugandan study, which reported that vitamin D was independently associated with increasing age^48^. However, when a correlation analysis was performed, a weak positive association was found. This association was statistically significant when a subanalysis was performed between age and TB patients alone. There is an increased risk of vitamin D deficiency with increasing age due to an inadequate diet with vitamin D, increased adiposity, and poor cutaneous handling of vitamin D metabolism due to fewer outdoor activities^49^. On the other hand, DBP, which is the major transporter of vitamin D, is affected by age.

The subanalysis by gender did not show a significant difference in vitamin D levels between males and females, although females had higher levels than males. However, none of the two groups had sufficient vitamin D levels. This finding is different from a study from Pakistan that found lower vitamin D levels in females than in males^42^. They attributed this to poor sunshine exposure of the female.

The high prevalence of hypovitaminosis D in all the study participant groups of active TB patients, LTBI, and those without TB infection is unique in that very few individuals had optimal vitamin D levels yet accordingly, Uganda has sunshine throughout the year and as determined by the sunshine exposure assessment tool of this study at least 42.0% participants reported having daily sunshine exposure between 1 and 7 h daily.

In general, regarding the high prevalence of hypovitaminosis D in African countries with high sunshine exposure, consideration of the alternative pathways of vitamin D3 metabolism could provide clear information on vitamin D status. Recent research by Slominski et al. provided evidence of a novel pathway of vitamin D3 metabolism involving cytochrome p450 enzymes such as CYP27B1, whose byproducts are biologically active metabolites^44^. Further follow-up research indicates that these pathways and their intermediate products have a physiological role evidenced by the presence of 22(OH)D3 and 20(OH)D3 in plasma, which was approximately 15 -30 times lower than that of 25(OH)D3^45^. This study suggests the inclusion of CYP11A in addition to 25(OH)D3 in the assessment of vitamin D hypovitaminosis.

On the other hand, the vitamin D nutritional assessment showed that the TB patient’s dietary intake hardly had foods that contain vitamin D. The earlier Ugandan study and another from Malawi that showed a high prevalence of vitamin D deficiency and hypovitaminosis D among hospitalized TB patients could probably have had poor nutritional status and long indoor behavior^48,50^.

No substantial conclusions have been made from previous studies on the cause-effect relationship of vitamin D status and TB disease; therefore, this remains to be explored using suitable study designs. According to Friis et al.^32^, vitamin D analysis is usually performed after the diagnosis of TB disease and may therefore not rule out vitamin D deficiency as a risk factor for TB disease. However, due to the high prevalence of hypovitaminosis D in the present study, nutritional and sunshine exposure information could provide evidence on inferences on the hypothesis of the cause-effect relationship of vitamin D deficiency and TB disease.

Nutritional and sunshine exposure data were not available for individuals and those without TB infection. Therefore, we were not able to assess this from those two groups. Our sample size was dependent on the amount of sample available; therefore, we had few control samples in the study. We did not measure other micronutrients or BMI to assess nutritional status.

The strength of our study is based on the fact that we selected household contacts with LTBI and those without infection who had been exposed to TB; therefore, the comparison was not biased.

In conclusion, we found a high proportion of hypovitaminosis D among this cohort of active TB patients, LTBI individuals, and those without TB infection with severe vitamin D deficiency in both TB patients and those without TB infection. Furthermore, vitamin D levels were significantly lower among TB patients than among LTBI individuals and those without TB infection. Individuals with low vitamin D levels have a high risk of TB disease progression. We, therefore, suggest that vitamin D levels be screened among household contacts to improve vitamin D status. Future studies are required to assess 25(OH)D with the inclusion of the cytochrome enzymes indicated in the alternative pathways of vitamin D3 as a better assessment of hypovitaminosis D.

## Methods

### Ethical considerations

This study was conducted according to the Helsinki Declaration regulations. Approval was sought from Makerere University SBS HDREC (#SBS-637), Research and Ethics Committee Mulago Hospital, Kiruddu Referral Hospital, National Council of Science and Technology (HS2639) to enroll PTB patients in this study. Furthermore, written informed consent was obtained from the active TB patients to participate in the study. Waiver of consent was obtained from Makerere University SBS HDREC (#SBS-637) to use the KTB samples after authorization from the KTB Principal Investigator. Details of the ethical approvals of the KTB study are found elsewhere^51^.

### Sample size, study design, and study population

A comparative cross-sectional study of pulmonary TB patients from the outpatient department of Kiruddu Hospital and retrieved samples of LTBI, (QFN^+^TST^+^) household contact with their controls (QFN^−^TST^−^) from the KTB study giving a sample size of 148 was perfomed. The study participants were recruited for the analysis of free and bioavailable vitamin D and cathelicidin  levels. Newly diagnosed positive Xpert MTB/Rif pulmona active TB patients aged 15–65 years were consecutively recruited between July 2019 and August 2020. Details of the KTB study have been described previously^51^. Total vitamin D analysis was required to categorize the vitamin D status of participants. Of the 148 participants, 105 met the inclusion criteria and were purposively selected for this study. The inclusion criteria were a 100 µl serum sample.

### Assessment of vitamin D diet intake and sunshine exposure

An administered self-reported frequency questionnaire on vitamin D intake adapted from a previous study that designed a tool for data converting data from a food frequency questionnaire to nutrient food values^52^. Sunshine exposure was assessed by asking the participants the length of time in hours they spent in the sun, which was adapted from another previous study^53^.

### Estimation of total 25(OH) D

Samples were collected in a plain tube and centrifuged to obtain serum, which was stored at − 80 °C before analysis. The analysis of vitamin D levels was performed by electrochemiluminescence using an Elecsys vitamin D3 assay according to the manufacturer’s instructions. The assay results were determined using a calibration curve, which is an instrument-specifically generated curve, by 2-point calibration and a master curve provided by the reagent barcode or e-barcoded. Vitamin D levels were categorized as deficiency ≤ 20.0 ng/ml, insufficiency = 21–29 ng/ml, sufficient ≥ 30 ng/ml and severe deficiency ≤ 10.0 ng/ml.

### Statistical analysis

Data were analysed using STATA software (Stata Corp. STATA version 16.0, College Station, Texas, USA. Data were tested for normality of distribution with the Shapiro–Wilk, Anderson–Darling, D'Agostino & Pearson test, and Kolmogorov–Smirnov tests. Continuous data were summarized into medians and IQR, confidence interval (CI) at 95%, and alpha of p < 0.05 was considered significant and a power of 80%. Categorical variables were summarized as frequencies, n (%). The Mann–Whitney U test was used for the analysis of two variables, the Kruskal–Wallis test was used for 3 or more categories for continuous variables, and Fischer's exact test was used for categorical variables. Spearman’s correlation test was used for correlations of vitamin D and age.

## Data Availability

All data and reagents are available on request by the corresponding author.
